# Extending the lore of curcumin as dipteran Butyrylcholine esterase (BChE) inhibitor: A holistic molecular interplay assessment

**DOI:** 10.1371/journal.pone.0269036

**Published:** 2022-05-26

**Authors:** Priyashi Rao, Dweipayan Goswami, Rakesh M. Rawal

**Affiliations:** 1 Department of Biochemistry & Forensic Science, University School of Sciences, Gujarat University, Ahmedabad, Gujarat, India; 2 Department of Microbiology & Biotechnology, University School of Sciences, Gujarat University, Ahmedabad, Gujarat, India; 3 Department of Life science, University School of Sciences, Gujarat University, Ahmedabad, Gujarat, India; National Cancer Institute at Frederick, UNITED STATES

## Abstract

Since its origin, the emergence of vector-borne infections has taken a toll on incalculable human lives. The use of chemical insecticides is one of the early known methods of vector control and although their use is still a prevalent way to combat insect population sadly the perils of insects related transmission still persists. Most commonly, the existing insecticides face the wrath of getting resisted repeatedly, paying way to develop resilient, efficient, and cost-effective natural insecticides. In this study, computational screening was performed using homology modelling, E-pharmacophore feature mapping, molecular docking, Density Function Theory (DFT) assessment, Molecular mechanics generalized Born surface area (MM-GBSA) based binding free energy calculations and Molecular Dynamics (MD) simulation to identify a potential lead phytochemical out of a manually curated library from published literature. The protein target used under this study is insect Butyrylcholine esterase (BChE). Additionally, *in vitro* insect (*Aedes aegypti*) BChE inhibition assay was also performed with the top phytochemical identified from *in silico* assessments. Our research highlights that curcumin leads to inhibition of enzyme BChE of *Ae*. *aegypti*. The identified mode of action of curcumin as an insect BChE inhibitor indicates the possibility of its use as an environment friendly and natural futuristic insecticide.

## 1. Introduction

Vector borne diseases (VBD) are a clan of diseases affecting over 80% of the world’s population residing in tropics and subtropics [1, 2]. The menace of dengue, chikungunya, Japanese encephalitis, malaria, yellow fever, filariasis, zika virus infection and others are known to exert a huge burden on global mortality rates [3, 4]. The transmission vectors responsible for majority of the VBD’s are mosquitoes like *Aedes*, *Anopheles* and *Culex* belonging to the family of *Culicidae* of Order Diptera, known for spreading the infections for entirety of their life span [5]. Recent reports suggest that the transmission dynamics is about to get worse with the rise in the phenomena of global warming [6]. Not only that, but the climatic changes might as well increase the re-emergence of VBD’s by boosting the rate of development of the infectious pathogen within the vector itself, thus putting the humanity at a greater risk [7].

Various strategies for vector control have been tailored over the course of these years taking into consideration the vector species; ranging from destroying the aquatic habitats, killing the adults using Indoor Residual Sprays (IRS), to the deployment of Insecticide treated bed Nets (ITN’s) for minimising vector-human contact [8]. Novel vector control strategy also employs the use of genetically modified *Wolbachia* as a measure to reduce incidences of VBD’s [9]. Contemplating all possible strategies, the use of chemical insecticides has been the most common method due to easy procurement, stock management and dispersion at civic breeding sites [10]. Amongst many, one of the reasons why the use of insecticide is frequent, is because they specifically interact and inhibit selective protein of the insect system. One such protein is Butyrylcholine esterase (BChE). This protein belongs to the class of Cholinesterase’s (ChE’s) which are the most important class of enzymes involved in neurotransmission in both vertebrates and invertebrates [11]. The principal enzyme of the same family is Acetylcholine esterase (AChE) which is also an absolute target of insecticides like carbamates and organophosphates [12, 13].

As incidences of resistance are bound to increase, and discovery of new target proteins and their antagonist is one of the priorities for proper vector management. Although not much is known about the specific functionalities of BChE in the insect system, but there are literary evidence of targeting BChE to control vector outgrowth. Multiple phytochemical fractions of plant *Calceolaria integrifolia* and *Calceolaria talcana* were observed to exhibit significant *in vitro* inhibitory effect of BChE on *Spodoptera frugiperda* (fall-armyworm), an insect belonging to the Order of Lepidoptera [14]. A recent study on dipteran *Aedes aegypti* reported about 80% to 100% of mosquitocidal activity at a concentration of 5μg/mosquito using macroalgae extracts of *Dictyota dichotoma* var. *intricata*. Metabolites of the same extract also revealed 50% BChE enzyme inhibition activity, suggesting the potentiality of macroalgae and its metabolites to be a natural product insecticide targeting the ChE’s [15].

This study is an extension of the work performed with an identical insecticidal target, AChE [16]. In this study, we have chosen BChE as a target and have evaluated the probability of its inhibition by phytochemicals. Till date there are several phytochemicals that are reported to possess larvicidal and insecticidal activity. With extensive literature survey we created a library of 70 phytochemicals that were reported to induce insect mortality. However, the underlying research gap lies in the fact of how these phytochemicals could induce insect mortality. Herein, we aim to identify a probable lead phytochemical that can interact with the protein target BChE. The first shortcoming for pursuing such study is the unavailability of tertiary protein structure of BChE from any of Diptera. Therefore, in current study we modelled a representative BChE protein making use of consensus sequence of mosquitoes. Thereafter, making use of computational studies involving ligand-based virtual screening, molecular mechanics, and Molecular Dynamic (MD) simulations, a top ranked phytochemical from the curated library was identified which may serve as a mosquito BChE inhibitor. Investigation of the geometrical and electric properties of the top ranked ligand was also done using Density Function Theory (DFT) calculation. The computational findings were further affirmed by performing *in vitro* enzyme inhibition assay from the larval tissue homogenate of *Aedes aegypti*. At every stage of experiments at both *in silico* and *in vitro* levels, appropriate known reference compounds were used as positive controls.

## 2. Materials and methods

### 2.1. Homology modelling

The sequences of BChE proteins were retrieved from UniProt [17] and GenPept. From UniProt the sequences retrieved were (i) *Aedes albopictus* BChE with accession id A0A023EWJ8 and (ii) *Aedes albopictus* BChE with accession id A0A023EUA0, while sequences from GenPept were (i) *Culex pipiens* BChE with accession id XP_039441597 and (ii) *Aedes aegypti* BChE with accession id XP_021698883.

As a 3D protein structure of BChE for dipteran species was not available, a representative model was built using consensus sequences from the above mentioned fasta files [18, 19]. All these sequences were aligned with Clustal Omega, and consensus sequences were extracted. From the consensus sequence so obtained, the protein crystallized BChE from PDB with having maximum sequence similarity was identified. The template used was PDB id “6QAA” of “Human Butyrylcholinesterase in complex with (S)-2-(butylamino)-N-(2-cycloheptylethyl)-3-(1H-indol-3-yl)propenamide”. Further, the template protein sequence and the query sequence (i.e., consensus sequence) were aligned using Clustal Omega and the missing gaps in the consensus sequence were filled with the analogous sequences from template protein, this modified query sequence so obtained was addressed as curated consensus sequence. Finally, the reference representative mosquito BChE model was developed by SWISS-MODEL using this curated consensus sequence as query sequence and target template as protein with PDB id “6QAA”. SWISS-MODEL server was used to compute the QMEAN and QMEANDisCo scores of the modelled protein [20, 21]. MolProbity v4.4 [22] was used to construct the Ramachandran plot of the modelled protein which provided the knowledge of the placement and stability of the individual amino acid of the protein. Lastly, the overall reliability and secondary quality check of the protein was performed using ERRAT analysis [23]. The co-ordinates of ligand binding site on BChE modelled protein were determined using CASTp 3.0 server (Computed Atlas of Surface Topography of proteins) [24] prior to molecular docking analysis.

### 2.2. Molecular docking and E-pharmacophore feature mapping for ligand screening

Docking assessments were performed in two sets where in the first set the modelled protein was docked with reference human BChE inhibitor drugs donepezil and rivastigmine at the protein cavity predicted by CASTp. These docked protein ligand complexes were then superimposed with the template protein “6QAA” to verify the correct binding site. For both the complexes BChE-donepezil and BChE-rivastigmine, the E-pharmacophore hypothesis were generated which were then used to screen the ligand library. The feature mapped phytochemicals were then hold-over for the second set of docking with BChE to identify top 5 phytochemicals.

Briefly, for docking assessment the modelled BChE protein was imported to Schrödinger Maestro and was prepared in ‘protein preparation wizard’ of Maestro. Here the protein was first pre-processed by adding hydrogens, converting selenomethionine to methionine and het states were generated by Epik for pH 7.0. In the next step of protein preparation, H-bond assignment was done using PROPKA for pH 7.0 for optimizing the protein. Once the protein was optimized, the restrained minimization of protein was done using OPLS-2005 (Optimized Kanhesia for Liquid Simulations) force field [25–27]. These tasks were all performed using the ‘protein preparation wizard’ of Schrödinger Maestro [28, 29]. Similarly, the optimized and minimized protein from the previous step was used for docking. Followed to which, the grid at the exact same co-ordinates as that of the cavity predicted by CASTp 3.0 was prepared with the box of the size 13 Å x 13 Å x 13 Å using receptor grid generation feature of Glide module in Schrödinger Maestro. For docking, the output file of (i) receptor grid generation and (2) prepared minimized ligands were imported in the ‘ligand docking’ window of Glide module in Schrödinger Maestro. Under the settings, the precision of docking was set as ‘Extra Precision (XP)’, Ligand sampling was set as ‘flexible’ and the Epik state penalties were added to docking score. The output was set to show only the best pose. The entire docking was performed using Glide module of Schrödinger Maestro [28, 29]. The output file of docking was used for performing MM-GBSA assessment.

The E-Pharmacophore method was employed to achieve the advantages of both ligand- and structure-based approaches of generating energetically optimized, structure-based pharmacophores to rapidly screen phytochemicals. The BChE-donepezil and BChE-rivastigmine were imported simultaneously into Maestro workspace and the E-pharmacophore hypothesis were developed through the ‘receptor-ligand complex’ of ‘develop pharmacophore model’ wizard in Phase module of Schrödinger Maestro. All seven (7) possible features were included in developing the individual hypothesis for each receptor-ligand complex. These hypothesises where collectively used to screen the ligand data set and for this, ‘ligand based screening’ wizard of Phase module of Schrödinger Maestro was used.

### 2.3. MM-GBSA calculations

Molecular mechanics generalized Born surface area (MM-GBSA) calculation was used to calculate the binding free energy change [30–32] using the Prime wizard of Maestro (Schrödinger Release 2017–4). Binding energy for each receptor-ligand complex was determined by using OPLS-2005 force field. Equation employed for free energy calculation is as follows:

ΔGBind=ΔEMM+ΔGSolv+ΔGSA (1)

Here, ΔEMM represents the variation between the minimized energy of the receptor–ligand complexes; ΔGSolv represents the variation between the GBSA solvation energy of the receptor–ligand complexes and the sum of the solvation energies for the protein and ligand. ΔGSA contains some of the surface area energies in the protein and ligand and the difference in the surface area energies for the complexes.

### 2.4. Density function theory

The optimal molecular geometry, charge distribution density and electrostatic properties of donepezil, rivastigmine and curcumin were analysed in gas phase using Density Functional Theory (DFT). Structures were minimised using the exact exchange function of Becke-3-parameter, Lee–Yang–Parr (B3LYP) method with the basis set. Optimisation was achieved using Gaussian 9 software [33]. Representation of Highest Occupied Molecular Orbital (HOMO) and Lowest Unoccupied Molecular Orbital (LUMO) was performed with the checkpoint files in GaussView 6 software [34].

### 2.5. MD simulations

Protein-ligand complexes possess dynamic characteristics and therefore analysing their movements at the atomistic level utilising MD simulation becomes essential in understanding the key physicochemical phenomena. Desmond (Schrödinger Release 2018–4) was used to perform simulation of BChE in the presence of the top lead phytochemical. To ensure the accuracy of MD simulation results, BChE-donepezil and BChE-rivastigmine complexes were used as a reference set. Docked complexes were prepared for MD simulation using ‘protein preparation wizard’ to ensure of pre-simulation protein relaxation. Briefly, the parameters for simulation involved a solvent model- TIP3P; with orthorhombic box constituting 13Å buffer space around the periphery of protein. Neutralisation was performed with the placement of Na^+^ ions and a salt concentration of 0.15 M Na^+^ and Cl^-^ counter ions to simulate the background salt at physiological conditions. Steepest descent energy minimization was performed, and the simulation was proceeded for 50 ns with NPT (constant Number of particles, Pressure, and Temperature) with 300 K and 1.01 bar, constant volume, smooth Particle-Mesh-Ewald (PME) technique. For the simulation time of 50ns, the energy recording interval was set at 1.2 ps and simulation trajectories recording interval was set at every 9.6 ps for each of the docked complexes. On completion of simulation, ‘simulation interaction diagram’ wizard of Desmond package was used to evaluate the trajectories for Root Mean Square Deviation (RMSD), Root Means Square Fluctuation (RMSF) and ligand-protein contact profiles.

### 2.6. Mosquito rearing

Test organism *Ae*. *aegypti* eggs were obtained from ICMR-National Institute of Malaria Research (NIMR) Field Unit (FU) Civil Hospital, Nadiad, Gujarat. Briefly, in a plastic container, a filter paper containing the mosquito eggs was placed with 500 ml distilled water for them to hatch in subsequent days. Water in the container was changed daily. The food source, temperature, photoperiod, water, and relative humidity are crucial parameters affecting the mosquito development at various stages of its life cycle. The mixture of yeast extract: dog biscuit: 10% sucrose solution, in the ratio of 1:3:1 constituted as food at the developmental stage which was administered twice a day. The temperature and humidity was maintained at 27±2°C and 70±9% respectively as per the methodology followed in the published literature [35]. Once the eggs hatched in water, mosquito larvae were transferred into a larger glass beaker with 1000 ml distilled water and continued food supplementation for rearing. Late 3rd or early 4th instar stage of mosquito larvae were used for *in vitro* BChE inhibition assay.

### 2.7. Larval BChE enzyme assay

The *in vitro* insect BChE inhibition assay, the protocol of Ellman [36] was followed with minimal modifications and 4^th^ instar stage of mosquito larvae were used. Briefly, for insect BChE estimation assay, the typical reaction mixture was prepared of 25.00μl of enzyme (larval lysate) suspended in 3.00ml of phosphate buffer (0.1M, pH 8.0), 25.00μl of 0.01M DTNB (5,5’-dithiobis-(2-nitrobenzoic acid) and 20.00μl of 0.1M S-Butyrylthiocholine Iodide substrate. The blank for such a run consists of buffer, substrate and DTNB solution, while the control included every other reaction component except the substrate. For the inhibition assay, the reaction mixture also comprised of curcumin (ranging from concentration of 50–250μM). A reference inhibition assay was also performed with commercially available BChE inhibitor, donepezil and rivastigmine (ranging from concentration of 2–10μM). Curcumin was obtained from Sigma-Aldrich Co., India while rest of the reagents were procured from Sisco Research Laboratories Pvt. Ltd., India.

## 3. Results

### 3.1. Homology modelling of BChE representative of diptera

**Fig 1:**
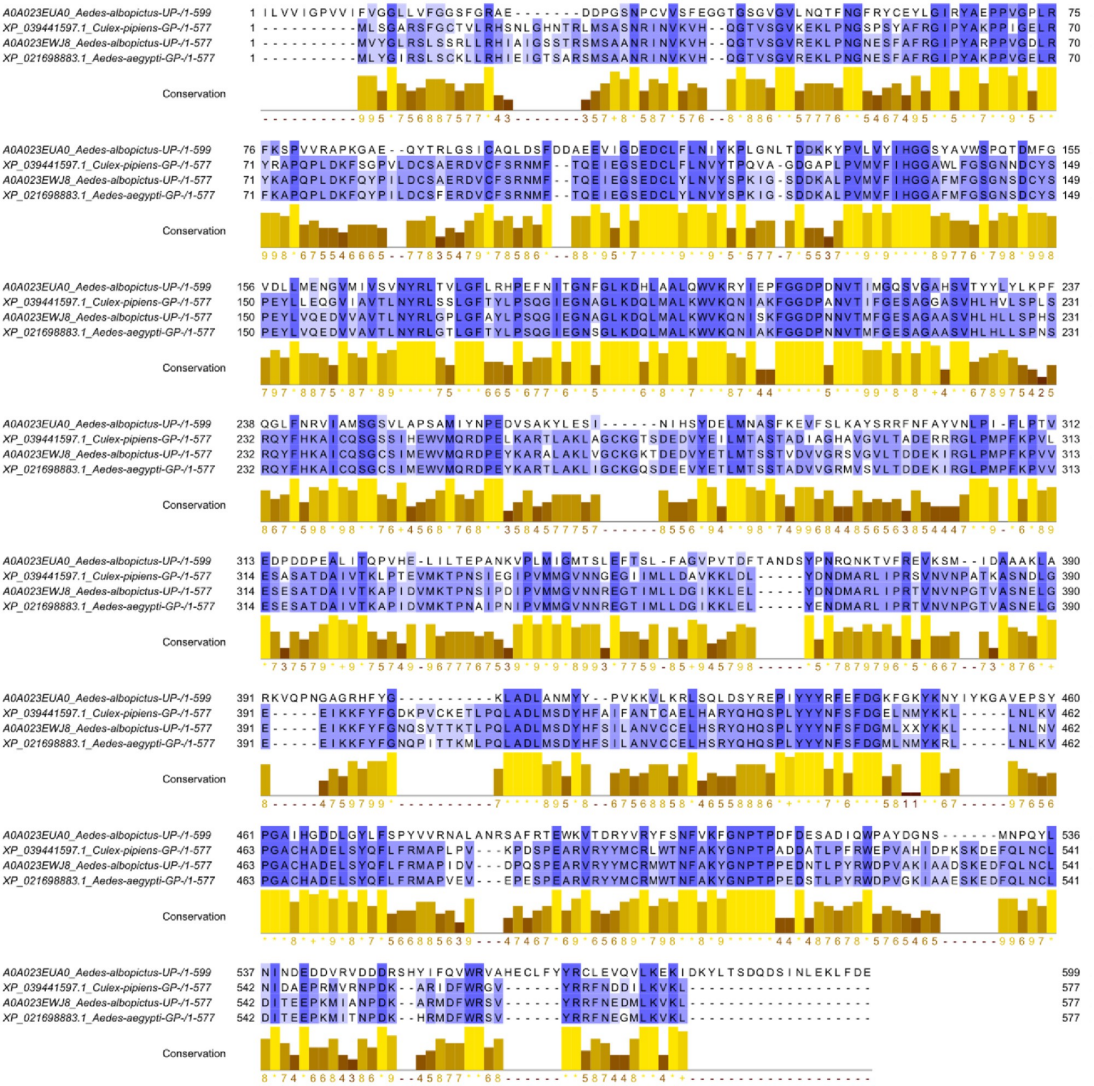
Multiple sequence alignment of BChE sequences from various mosquitoes and identified conserved regions.

**Fig 2:**
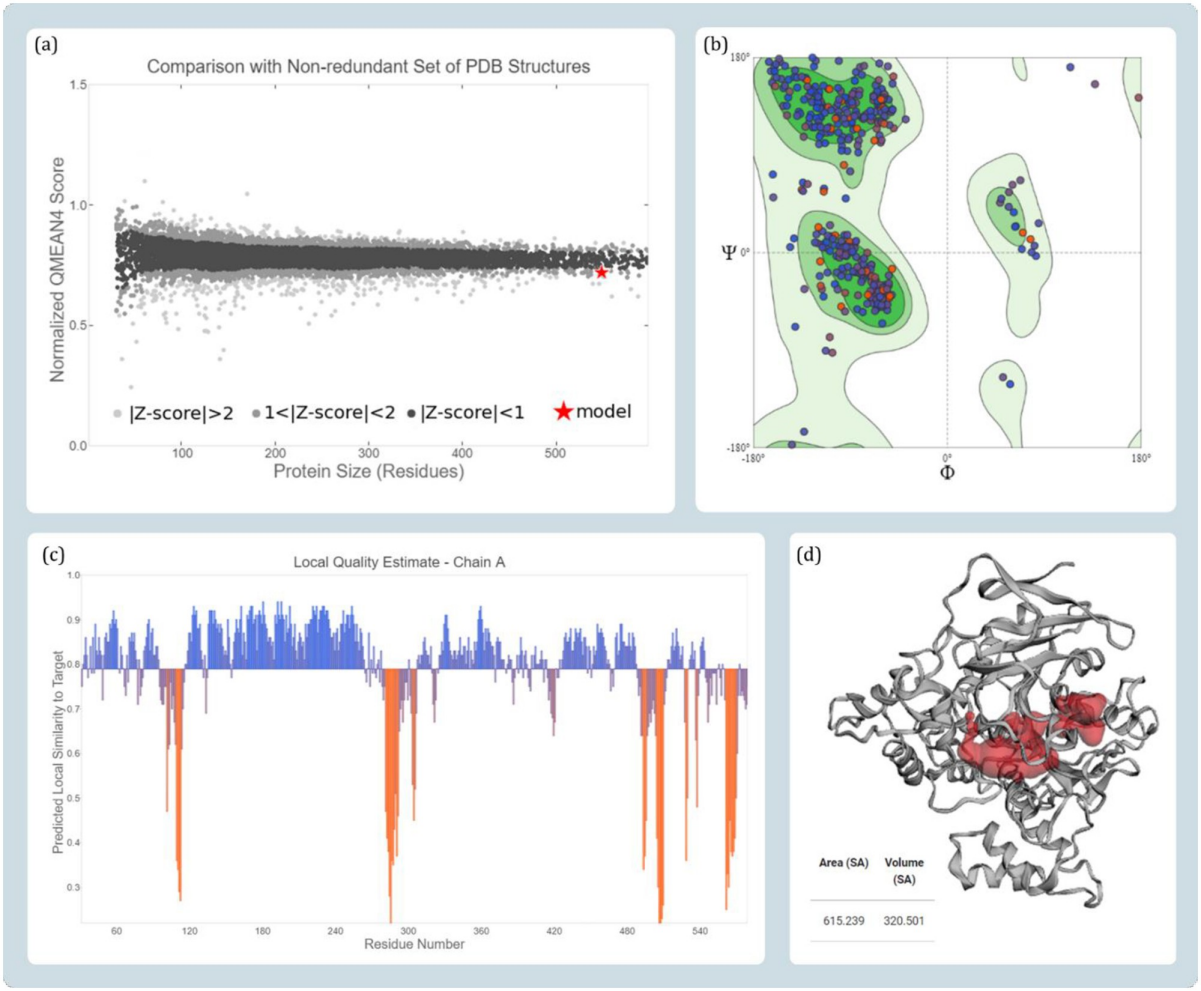
Quality estimate parameters for modelled BChE protein (a) comparison with non-redundant set of PDB structures (b) Ramachandran plot (c) local model quality estimate and (d) binding pocket identification of modelled protein predicted by CASTp 3.0.

The approach used in the current study is to make use of consensus sequence to construct the model as a reference representative mosquito dipteran BChE. For this the sequences from UniProt the sequences retrieved were (i) *Aedes albopictus* BChE with accession id A0A023EWJ8 and (ii) *Aedes albopictus* BChE with accession id A0A023EUA0, while sequences from GenPept were (i) *Culex pipiens* BChE with accession id XP_039441597 and (ii) *Aedes aegypti* BChE with accession id XP_021698883 as represented in Fig 1 were aligned using Clustal Omega and the conserved consensus sequence was extracted. This consensus sequence showed maximum similarity with the protein with id ‘6QAA’ from PDB. After filling he gaps in the consensus sequence with the analogous sequences from ‘6QAA’ the curated sequence was developed. The homology model was developed using this curated sequence with keeping template as ‘6QAA’. The curated consensus sequence and ‘6QAA’ showed 70% sequence similarity and the query coverage was 98%. Quality assessment parameters, (i) GMQE between 0 to 1 is considered as ideal, which for the modelled BChE was found to be 0.76, (ii) QMEAN Z-scores assessment value for model was found to be 0.79, where the value closer to 0 is considered to a marker of getter quality and the values lesser than -4.0 suggests the quality of model to be poor, (iii) MolProbity results of Ramachandran plot indicated 90.13% favoured residues, while 1.83% residues as Ramachandran outliers and the overall MolProbity score was found to be 1.89 (Fig 2).

### 3.2. Docking of modelled BChE with reference drugs

**Fig 3:**
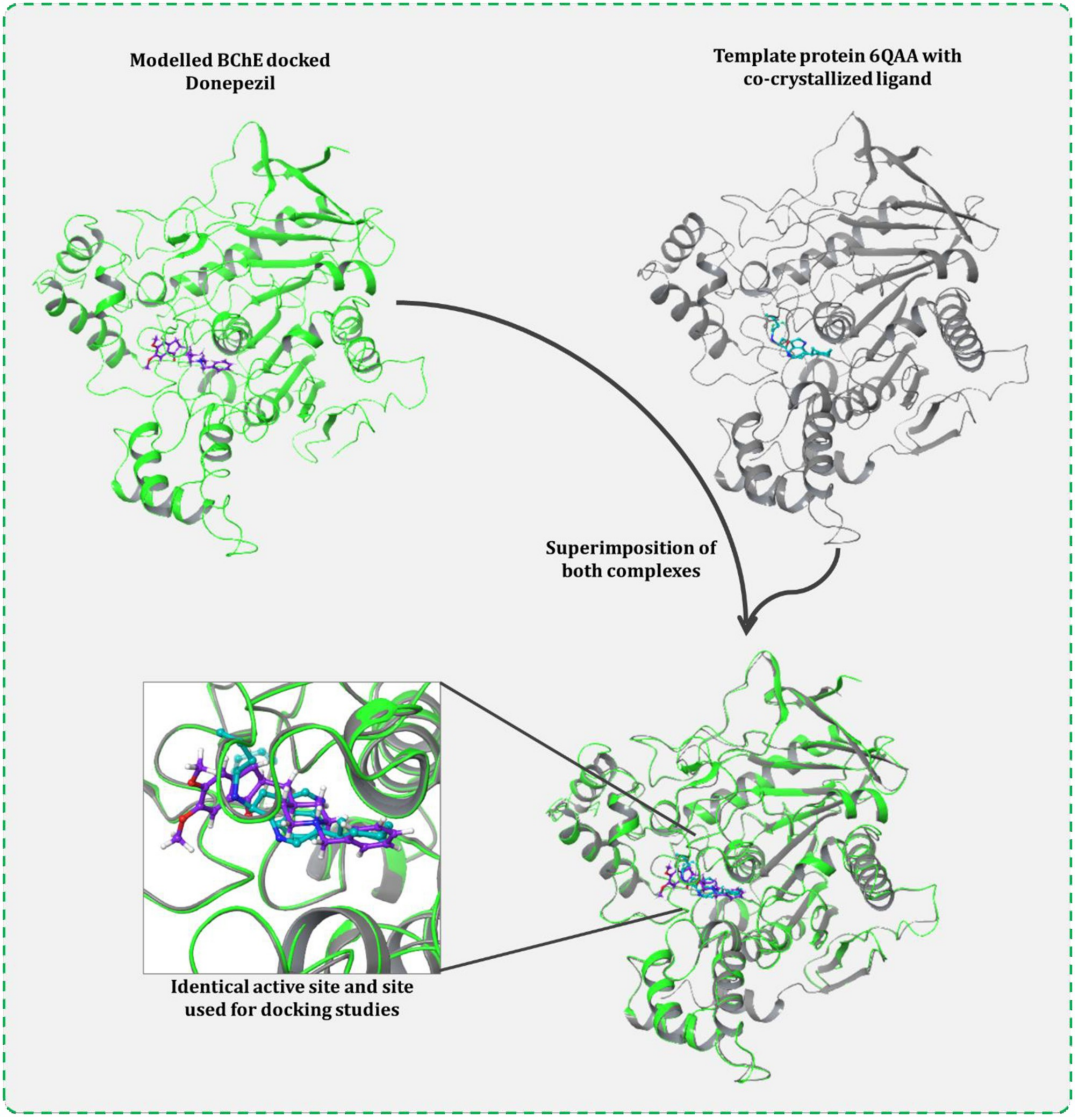
Validation of correct active site coordinated for docking, proved by identical binding of Donepezil with modelled BChE to that of native ligand of template protein (PDB id 6QAA). Superimposing both the protein, the active sites also get super imposed, and the respective ligand of proteins also gets superimposed at identical locus validating the coordinates of docking used for modelled BChE is apt.

**Fig 4:**
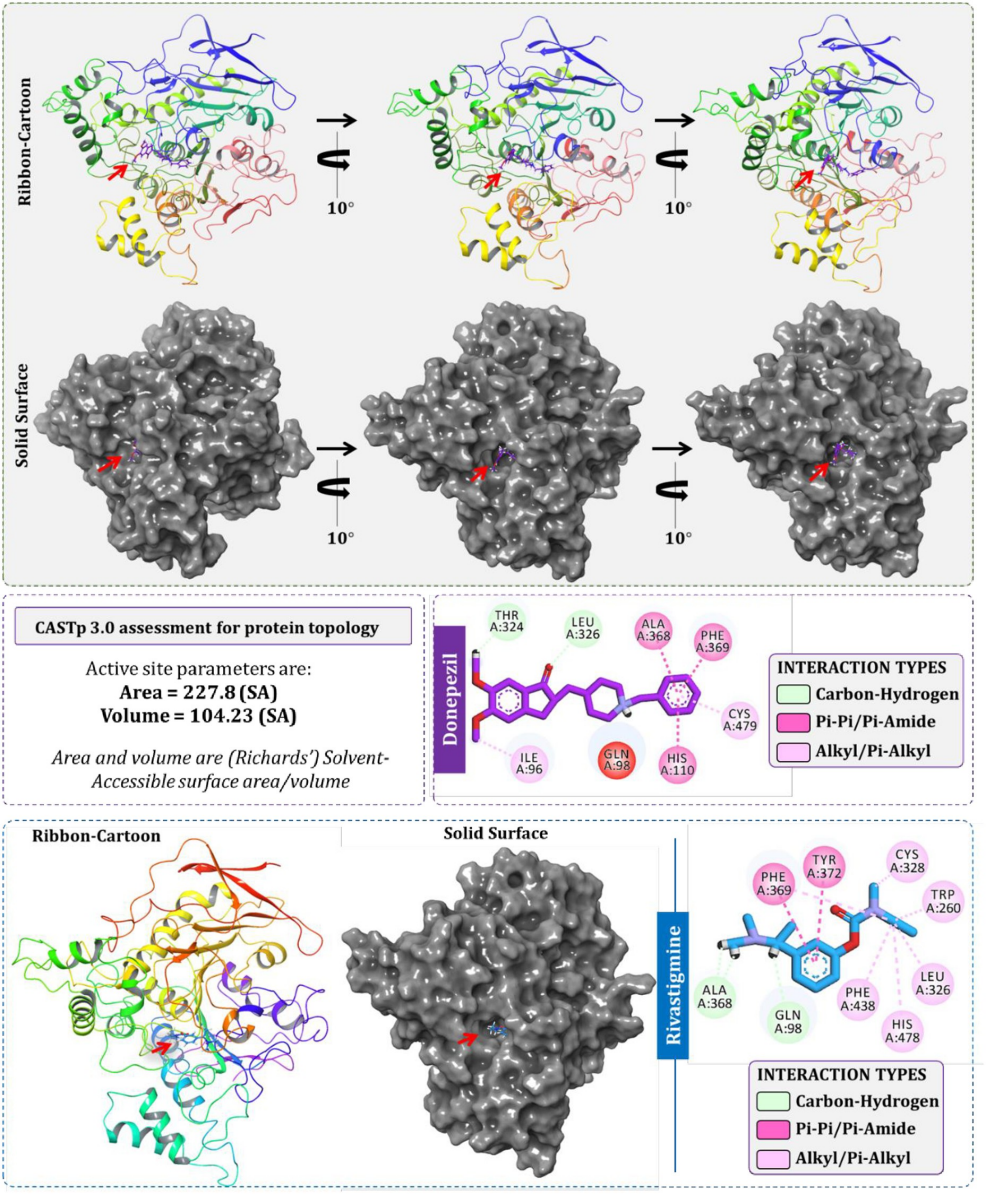
Molecular docking of donepezil and rivastigmine with modelled BChE at the identified active site with the help of CASTp 3.0.

The two drugs donepezil and rivastigmine are known BChE inhibitors and therefore they were used as a reference compound for primary docking studies. The active site of BChE was primarily predicted using CASTp 3.0 server. The coordinates of the cavity predicted by the server was used for docking of these reference drugs. The predicted protein cavity was found to be having the total area of 227.8 Richards’ accessible surface area (SA) and volume of 104.23 Richards’ accessible volume (SA) (Figs 2 and 4). On performing the docking both the drugs occupied the predicted binding cavity with decent binding scores (Table 1). To validate, the appropriate locus of drug binding with BChE, the docked complex was superimposed with the template protein PDB id ‘6QAA’ with co-crystallized ligand (S)-2-(butylamino)-N-(2-cycloheptylethyl)-3-(1H-indol-3-yl)propenamide. On superimposing both the protein, the active sites as well as the respective ligand of proteins got superimposed at identical locus. Thus, proving the coordinates of docking used for modelled BChE is fitting (Fig 3). Donepezil interacted with BChE by making dominant hydrophobic interactions where His110, Ala368, and Phe369 formed Pi-Pi interactions while Ile96 and Cys479 formed Pi-alkyl interactions. Additionally, Thr324, and Leu326 formed C-H bonds. Similarly, rivastigmine interacted with BChE by making dominant hydrophobic interactions with amino acid residues Phe369 and Tyr372 formed Pi-Pi interactions. Pi-alkyl interactions were formed with five different amino acids namely, Trp260, Cys328, Leu326, His478, and Phe438. Lastly, Gln98 and Ala368 formed C-H bonds (Fig 4).

**Table 1:** Docking and MM-GBSA energy profiles of control drugs and screened phytochemicals during their interaction with BChE.

Compound name	Docking	MM-GBSA (Gibbs free energy ΔG, and unit for the expressed values is kcal/mol)				
	Binding energy (kcal/mol)	ΔGBind	ΔGCoulomb	ΔGHbond	ΔGLipo	ΔGvdW
**Drugs (control)**						
Donepezil	-5.947	-59.479	-16.132	-0.372	-29.182	-48.71
Rivastigmine	-4.406	-36.637	-11.569	-0.561	-15.079	-32.884
**Phytochemicals (screened hits)**						
Curcumin	-8.773	-63.007	-25.573	-1.685	-23.51	-43.731
Desmethoxycurcumin	-8.187	-54.707	-14.885	-0.909	-19.526	-46.924
Gingerol	-7.271	-52.698	-22.596	-1.94	-21.313	-32.631
Asarinin	-5.846	-47.902	-7.335	-0.462	-20.014	-47.213
Capsaicin	-7.25	-46.833	-15.708	-1.674	-20.914	-43.269
Sesamin	-6.398	-46.467	-6.797	-0.424	-20.326	-46.069
6-Shogaol	-5.859	-43.425	-6.764	-0.321	-21.439	-43.863
Rosmarinic Acid	-10.022	-40.487	-8.133	-2.614	-24.15	-36.701
Piperine	-5.233	-39.608	-4.272	-0.21	-20.5	-37.995
Hesperetin	-7.232	-35.775	-7.694	-1.236	-10.514	-39.058
Zingiberene	-4.867	-29.753	-2.124	0	-20.178	-35.212
Betulinic acid	-3.541	-22.247	23.357	-1.158	-16.236	-33.354
Ursolic Acid	-3.261	-2.88	37.594	-0.97	-28.658	-18.629

ΔGBind—Binding energy, ΔGCoulomb—Coulomb energy, ΔGHbond—Hudrogen-bonding correction, ΔGLipo—Lipophilic energy, ΔGvdW—Van der Waals energy

### 3.3. E-Pharmacophore feature mapping-based ligand screening

**Fig 5:**
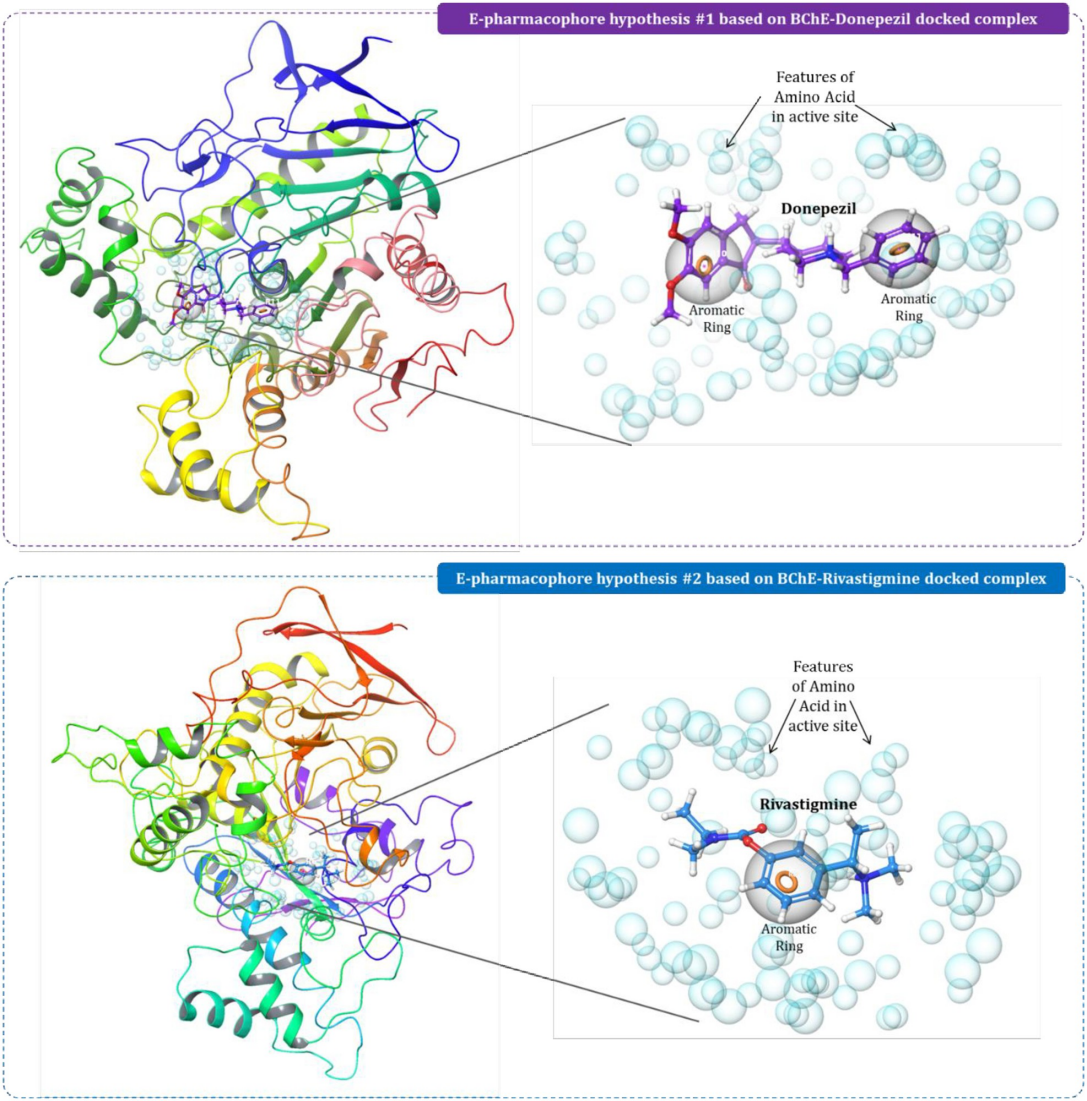
E-pharmacophore hypothesis of BChE-donepezil and BChE-rivastigmine complexes.

Using the docked complexes (i) BChE-donepezil and (ii) BChE-rivastigmine E-pharmacophore hypothesis was generated for each these complexes. For the BChE-donepezil, the important pharmacophore features identified were two aromatic rings found to be important for making hydrophobic interactions with the protein (Fig 5). While for BChE-rivastigmine complex, a similar pharmacophore feature was mapped with one aromatic ring (Fig 5). Ligand based screening of 70 phytochemicals was performed against both the generated hypothesis. In totality, 12 phytochemicals were filtered based on identical pharmacophore features which were then used for docking and MM-GBSA calculation.

### 3.4. Docking and MM-GBSA calculations of screened hits

**Fig 6:**
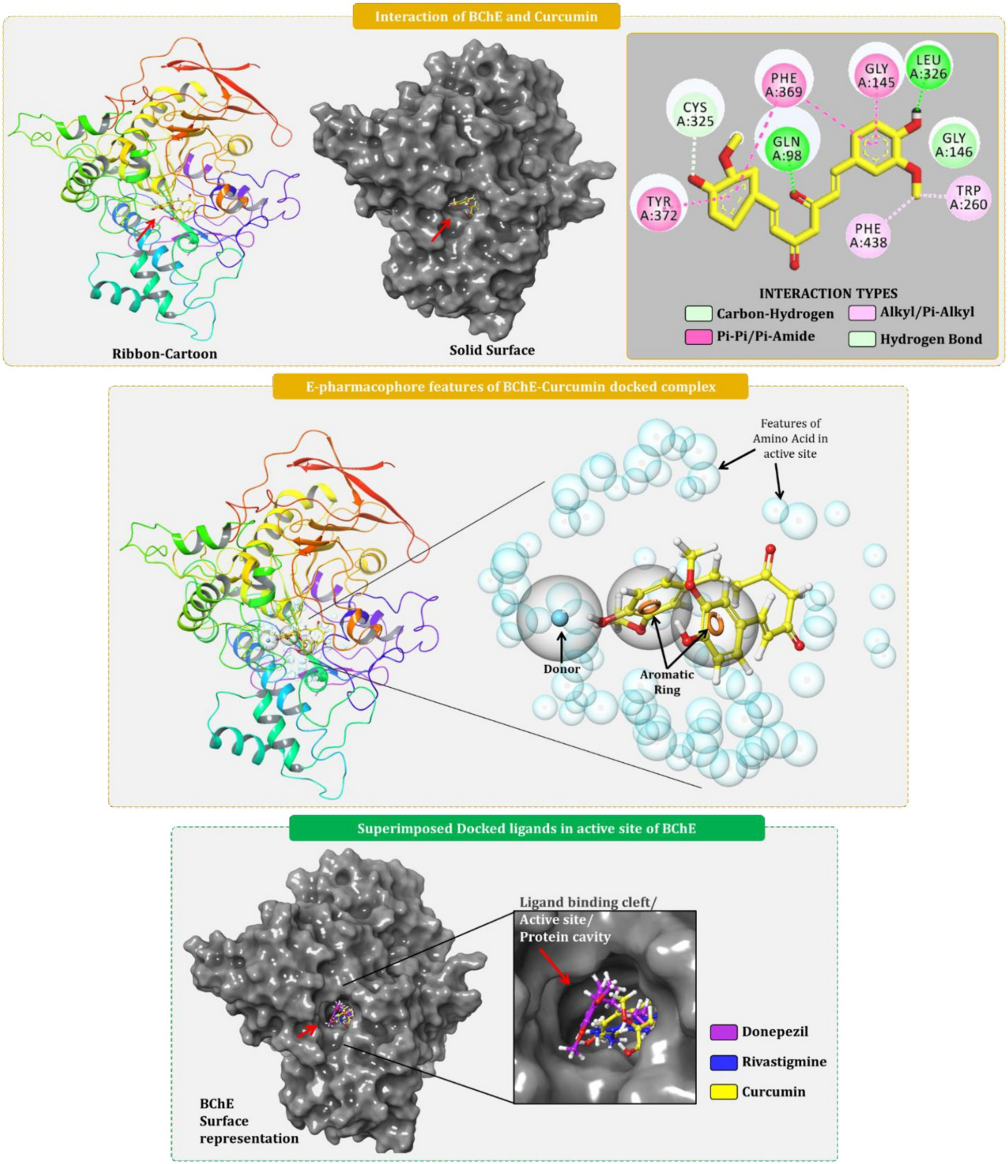
Docking assessment of curcumin with BChE and E-pharmacophore feature development of BChE-curcumin complex.

The 13 screened phytochemicals used for XP docking and MM-GBSA calculation were curcumin, desmethoxycurcumin, gingerol, asarinin, capsaicin, sesamin, 6-shogaol, rosmarinic acid, piperine, hesperetin, zingiberene, betulinic acid, and ursolic acid. 10 out of 13 compounds showed the binding energy better than rivastigmine (-4.406 kcal/mol) while 6 out of 13 compounds showed binding energy better than donepezil (-5.947 kcal/mol) (Table 1). Curcumin and desmethoxycurcumin showed exceptional binding energy of -8.773 kcal/mol and -8.187 kcal/mol respectively which was depicting that these compounds were much efficient in binding with BChE than the reference drugs. MM-GBSA assessment showed the binding free energy change (ΔGBind) of donepezil and rivastigmine -59.479 kcal/mol and -36.637 kcal/mol respectively. Curcumin and desmethoxycurcumin showed the ΔGBind of -63.007 kcal/mol and -54.707 kcal/mol. Curcumin showed better ΔGBind for both the reference drugs while desmethoxycurcumin had better ΔGBind than rivastigmine but inferior to donepezil. Thus, based on docking and MM-GBSA assessment, curcumin was a lead phytochemical identified. Like control drugs, curcumin was effectively able to interact with BChE by making hydrophobic interactions involving amino acids residues Gly145, Phe369, Tyr372 (Pi-Pi interactions); Trp260 and Phe438 (Pi-alkyl interactions); Cys325 (C-H bonds); while hydrophilic interactions with Gln98 and Leu326 (hydrogen bonds) (Fig 6). Moreover, the E-pharmacophore features of BChE-curcumin complex were identical to that of BChE- donepezil complex (Fig 6).

### 3.5. DFT assessment

**Fig 7:**
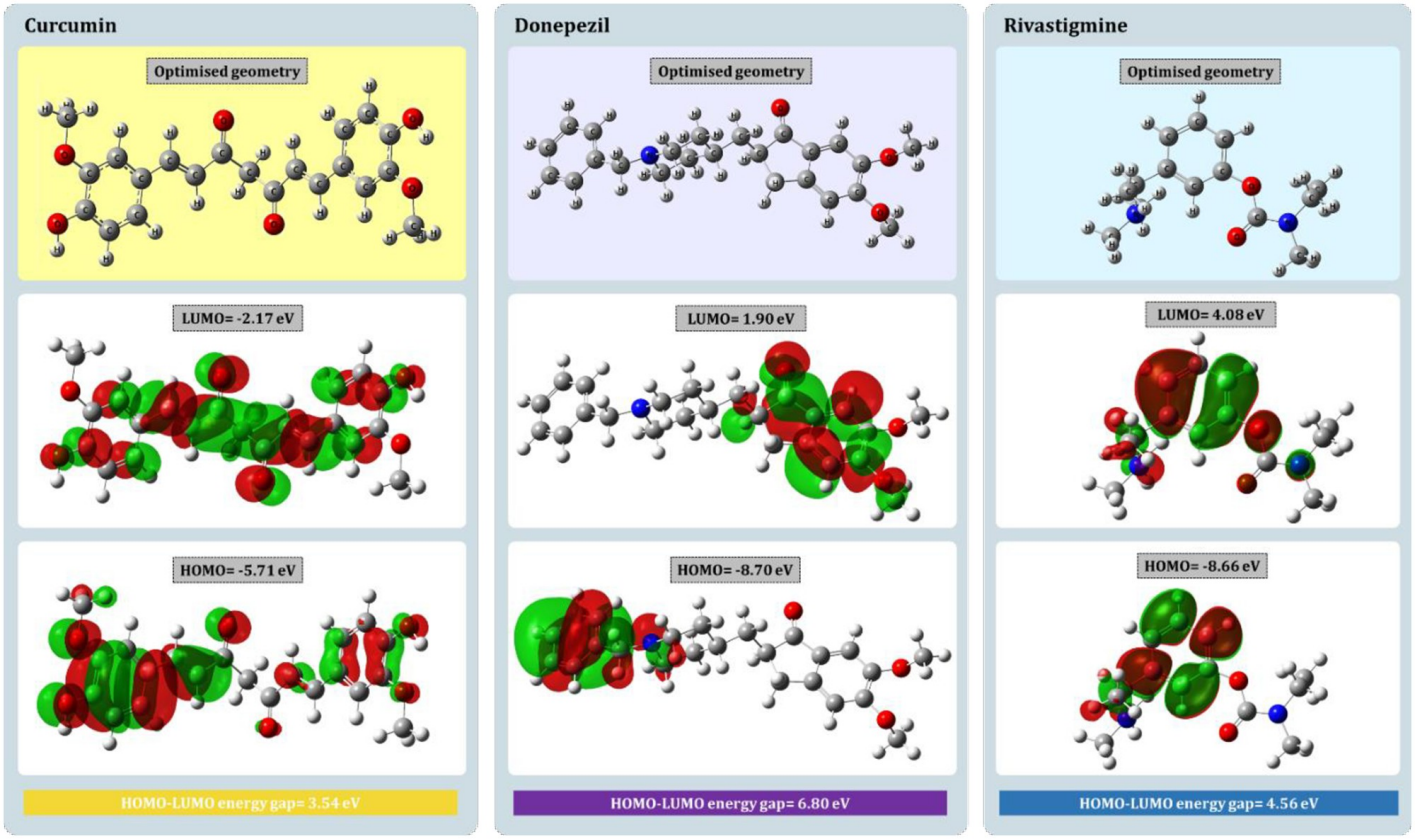
HOMO, LUMO and optimized geometry of curcumin, donepezil and rivastigmine obtained by DFT assessment.

The HOMO energy of a compound refers to its ability to give electrons while forming intermolecular complexes, meanwhile the LUMO energy refers to the compound’s ability to accept electrons from the neighbouring macromolecule. Also, the electronic excitation energy (difference in HOMO and LUMO energy of a molecule) is used to calculate the molecular stabilization and activity of the molecules. Smaller the energy gap, highly reactive and stable the compound is. In this case, HOMO, LUMO and optimized geometry of curcumin, donepezil and rivastigmine is represented in Fig 7. Firstly, mentioning the reference drugs, for donepezil the HOMO energy is -8.70eV while the LUMO energy is 1.90eV, corresponding the energy gap to be 6.80eV, whilst for rivastigmine the HOMO energy is -8.66eV while the LUMO energy is 4.08eV, attributing the energy gap of 4.56eV. The top lead phytochemical displays a rather smaller energy for HUMO to be of -5.71eV and LUMO to be -2.17eV, and the energy gap is calculated to be 3.54eV. This difference in electronic density between the enolic hydroxyl group and the carbonyl group was found to be a favourable centre for nucleophilic attack in curcumin by DFT analysis. This suggests that this area may have a role in enzymatic activity. The results of molecular docking also pointed to the establishment of hydrogen bonds involving amino acids. These regions ensure the formation of bonding, and it is one of the probable justifications for the effectiveness of the phytochemical, curcumin.

### 3.6. MD simulations

A 50 ns MD simulation were performed for the complexes of (i) BChE-curcumin (ii) BChE-donepezil and (iii) BChE-rivastigmine, to consolidate the findings of docking with respective ligand interaction of BChE. RMSD, RMSF and protein-ligand contact profiles with their interaction timeline and interaction types of all the MD trajectories were calculated. Curcumin was identified to be the best hit from docking and MM-GBSA assessment, so BChE-curcumin MD run was considered as the test while the MD runs of complexes BChE-donepezil and BChE-rivastigmine were considered as reference control.

**Fig 8:**
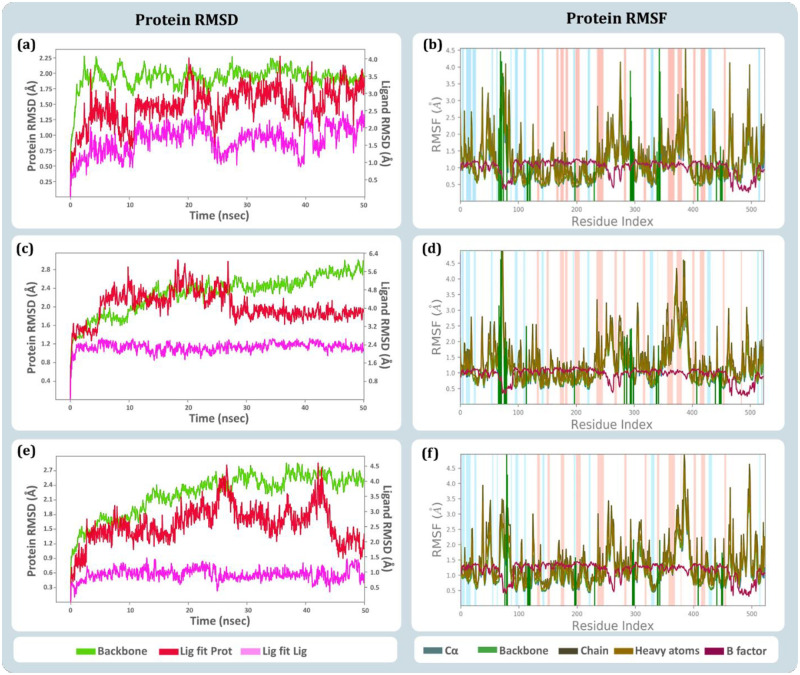
RMSD and RMSF profile obtained on performing 50 ns MD simulation of (a) RMSD of BChE-curcumin complex (b) RMSF of BChE1-curcumin complex (c) RMSD of BChE-donepezil complex (d) RMSF of BChE1-donepezil complex (e) RMSD of BChE-rivastigmine complex, and (f) RMSF of BChE-rivastigmine complex.

The RMSD of individual molecules in the protein ligand complex and their movements with respect to each other is depicted in Fig 8. The RMSD values in the left Y-axis denotes the value of protein and the right Y-axis represents the value of ligand. For the protein assessment only the value on the left Y-axis is to be seen where the values up to 3 Å is considered ideal, while the values above 3 Å represents the conformation change in the 3D architecture of the protein. Here, it was observed that the 3D architecture of BChE being stable in presence of all the three ligands curcumin (Fig 8a), donepezil (Fig 8c) and rivastigmine (Fig 8e), none of these complexes appear to be exceeding the protein RMSD beyond 2.9 Å. Additionally, Ligand RMSD represented in the right Y-axis, describes the movement of ligand with respect to the protein (Lig fit Prot). Also, the individualistic RMSD of ligand is described as ‘Lig fit Lig’ in the RMSD plots. The ‘Lig fit Prot’ values close to protein RMSD suggests equal movement of protein backbone and ligand. Ligands being much smaller in size than the protein often demonstrate the ‘Lig fit Prot’ values higher than protein RMSD. However, two to three folds higher values of ‘Lig fit Prot’ than that of protein RMSD suggests the ligand is changing poses and orientations in the protein cavity to attain stable spatial arrangement. In this study, the ‘Lig fit Prot’ values for all the three ligands does not exceed by two folds with respect to the corresponding protein RMSD values, suggesting that all the three ligands acquire stable conformation in the active site of BChE confirming the poses of ligands predicted by docking to be appropriate.

Further, RMSF plots of BChE-curcumin (Fig 8b), BChE-donepezil (Fig 8d) and BChE-rivastigmine (Fig 8f) suggests local changes along the protein chain and peaks on this plot indicate areas of the protein that fluctuate the most during the simulation. The α helices and β strands are usually more rigid shows less fluctuations while the tails of N and C terminals are flexible shows higher. The vertical green lines show the region of protein that interact with ligand, these regions show similarity in RMSF values while interacting with all the three different ligands. This suggests the binding of ligands at the active cleft has allowed α helices and β strands is identical for all the three ligands and RMSF trend represented by BChE while interacting with all the three ligands is also similar.

**Fig 9:**
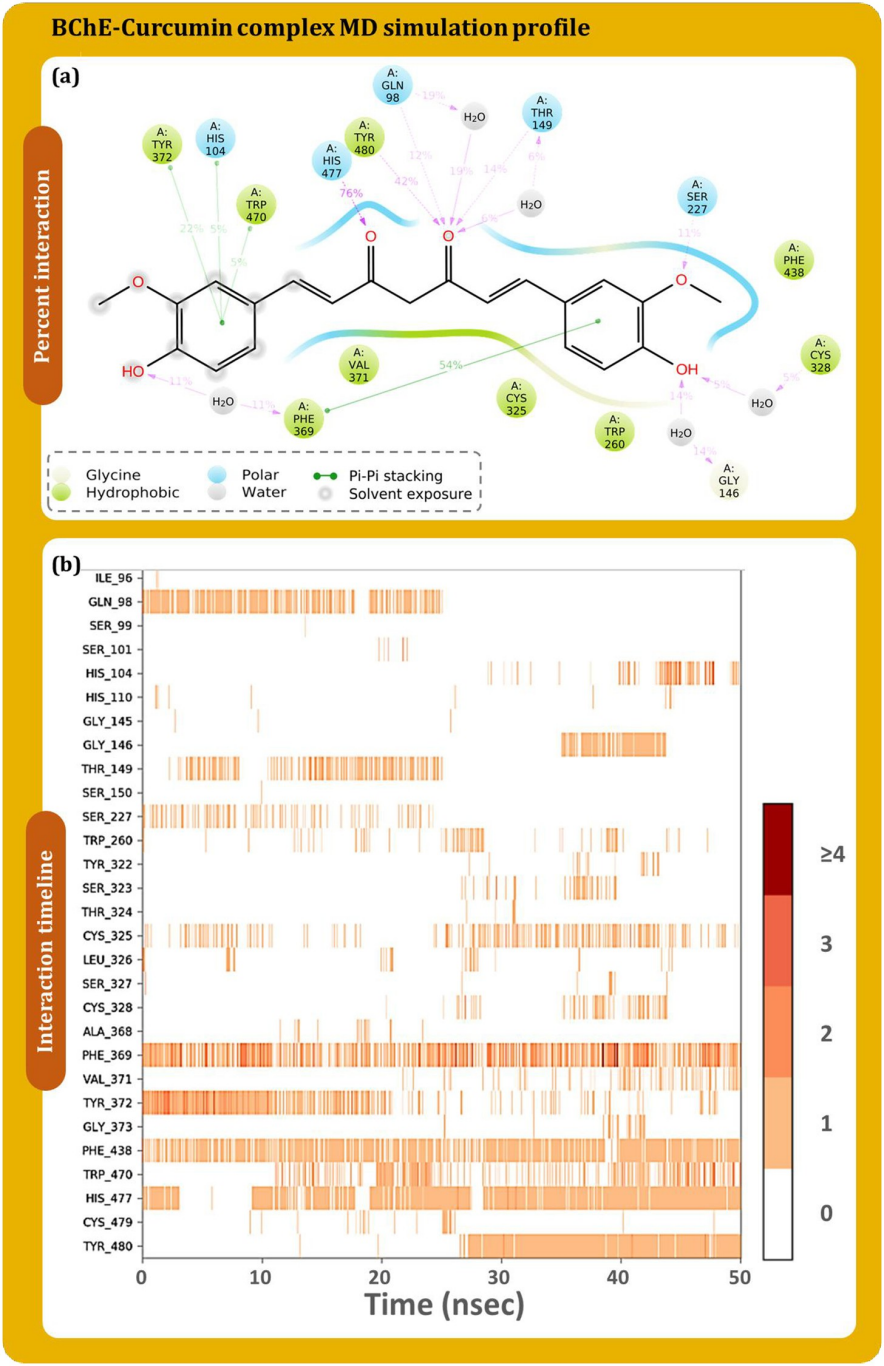
BChE-curcumin (a) percent interaction profile and (b) interaction timeline profile obtained on performing 50 ns MD simulation.

**Fig 10:**
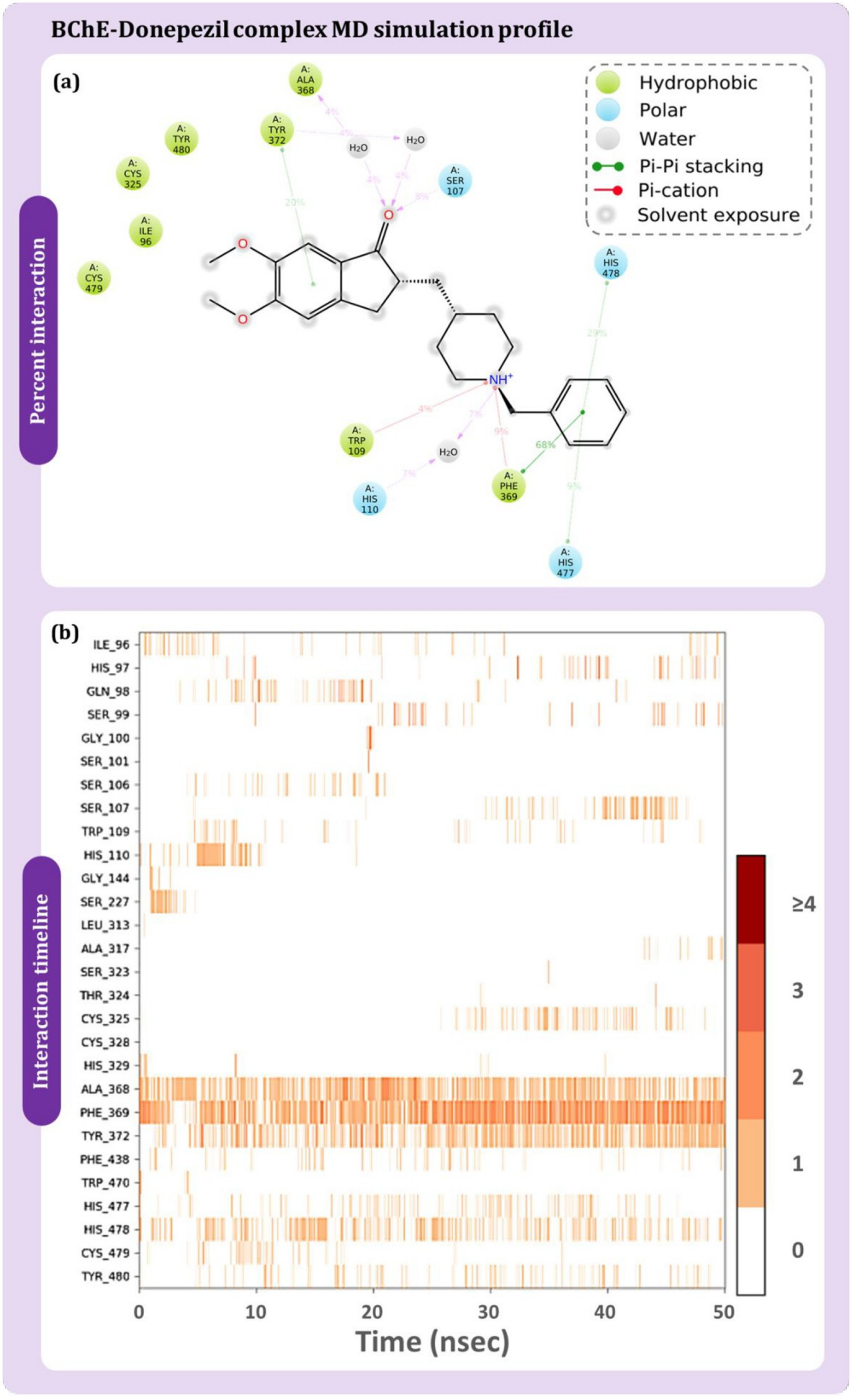
BChE-donepezil (a) percent interaction profile and (b) interaction timeline profile obtained on performing 50 ns MD simulation.

**Fig 11:**
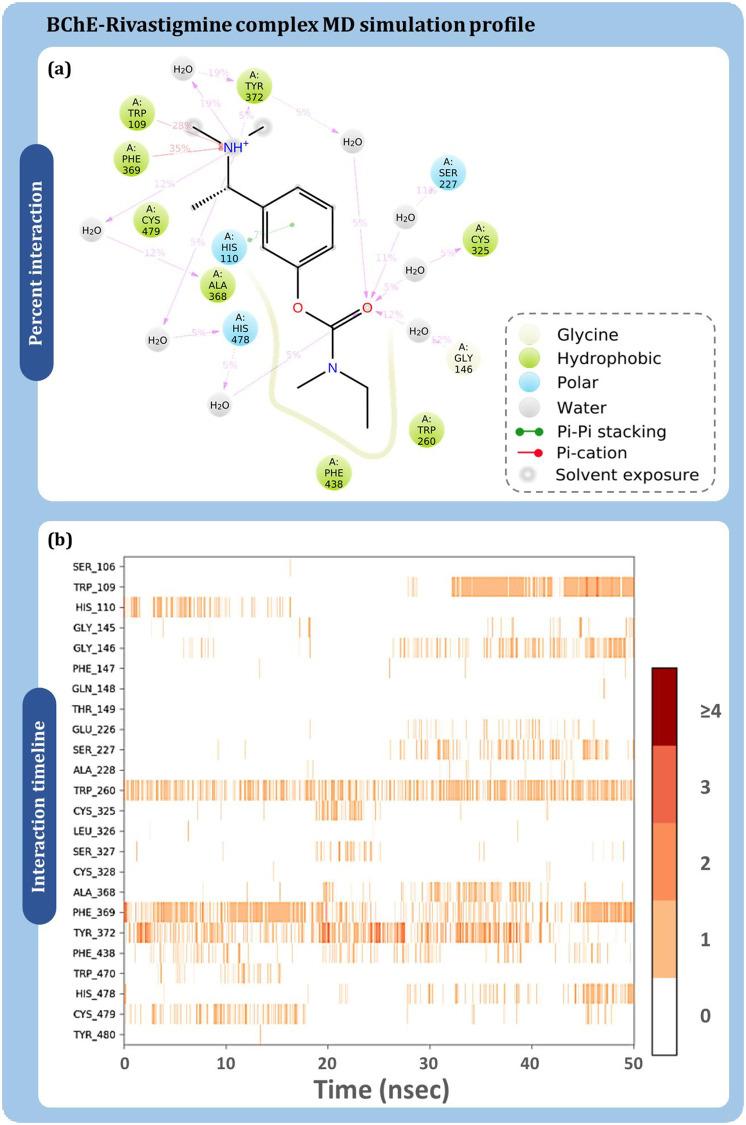
BChE-rivastigmine (a) percent interaction profile and (b) interaction timeline profile obtained on performing 50 ns MD simulation.

The protein-ligand contact timeline and their percent interaction occurring between BChE and curcumin are described in Fig 9. Fig 9a shows that His477 having interaction by 76% of the time during MD simulation followed by Phe369 by 54%, Tyr480 by 42%, Tyr372 by 22%. Fig 9b is a timeline plot of the 50ns interaction and demonstrates Phe438 to strongly interact with curcumin. There are other amino acids which also plays a crucial role in interaction with curcumin and their interaction percent with respect to time is represented in Fig 9. Similarly, Fig 10 shows the interaction profile of donepezil interaction with BChE. Ala368, Phe369, Tyr372, and His478 are shown to frequently interact with BChE, where Phe369 is shown to interact the most with 68% of simulation time by making pi-pi stacking while additionally making pi-cation interaction with 9%. Similarly, for rivastigmine (Fig 11), Phe369 interacts by 35% and Trp109 by 28% as top directly interacting amino acids (Fig 11a). Amino acid interaction timeline (Fig 11b) shows His110, Trp260, Phe369, Tyr372, His478 and Cys479 to frequently interact with BChE.

**Fig 12:**
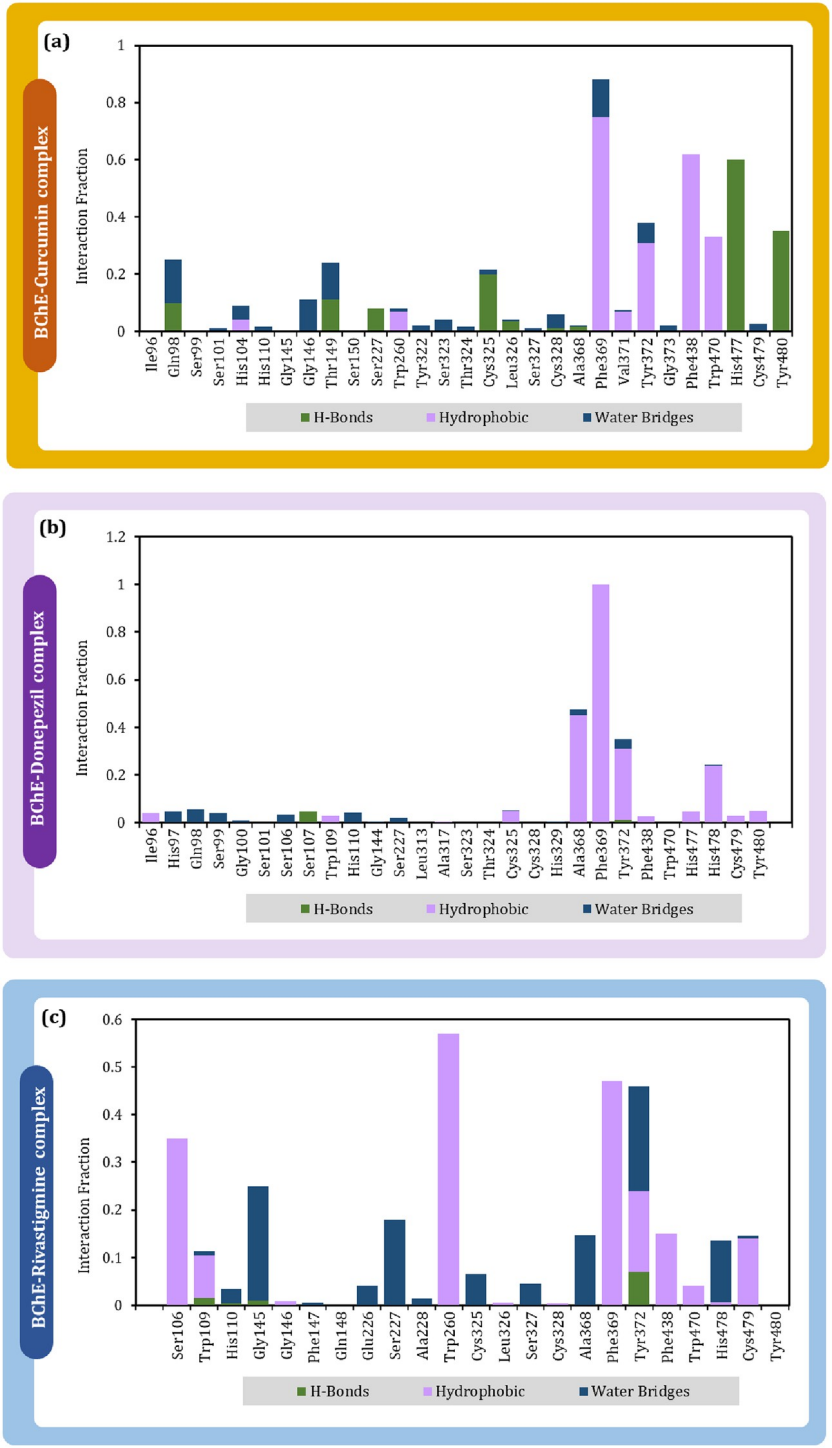
Interaction fraction profile with interaction types obtained on interaction of (a) curcumin (b) donepezil and (c) rivastigmine with BChE during 50 ns MD simulation.

The consolidated interaction types exhibited by all the ligands is represented in Fig 12, which are in form of hydrogen bonds, hydrophobic interactions, and water bridges. Interaction of curcumin (Fig 12a), donepezil (Fig 12b) and rivastigmine (Fig 12c) with BChE is shown. Phe369 is found to be the common interacting residue for all the three ligands. The interaction fraction of curcumin was found to be much higher with BChE with in concurrence with the reference drugs. This shows curcumin to exhibit at par or even better interaction with BChE than by reference drugs.

### 3.7. Larval BChE enzyme assay

**Fig 13:**
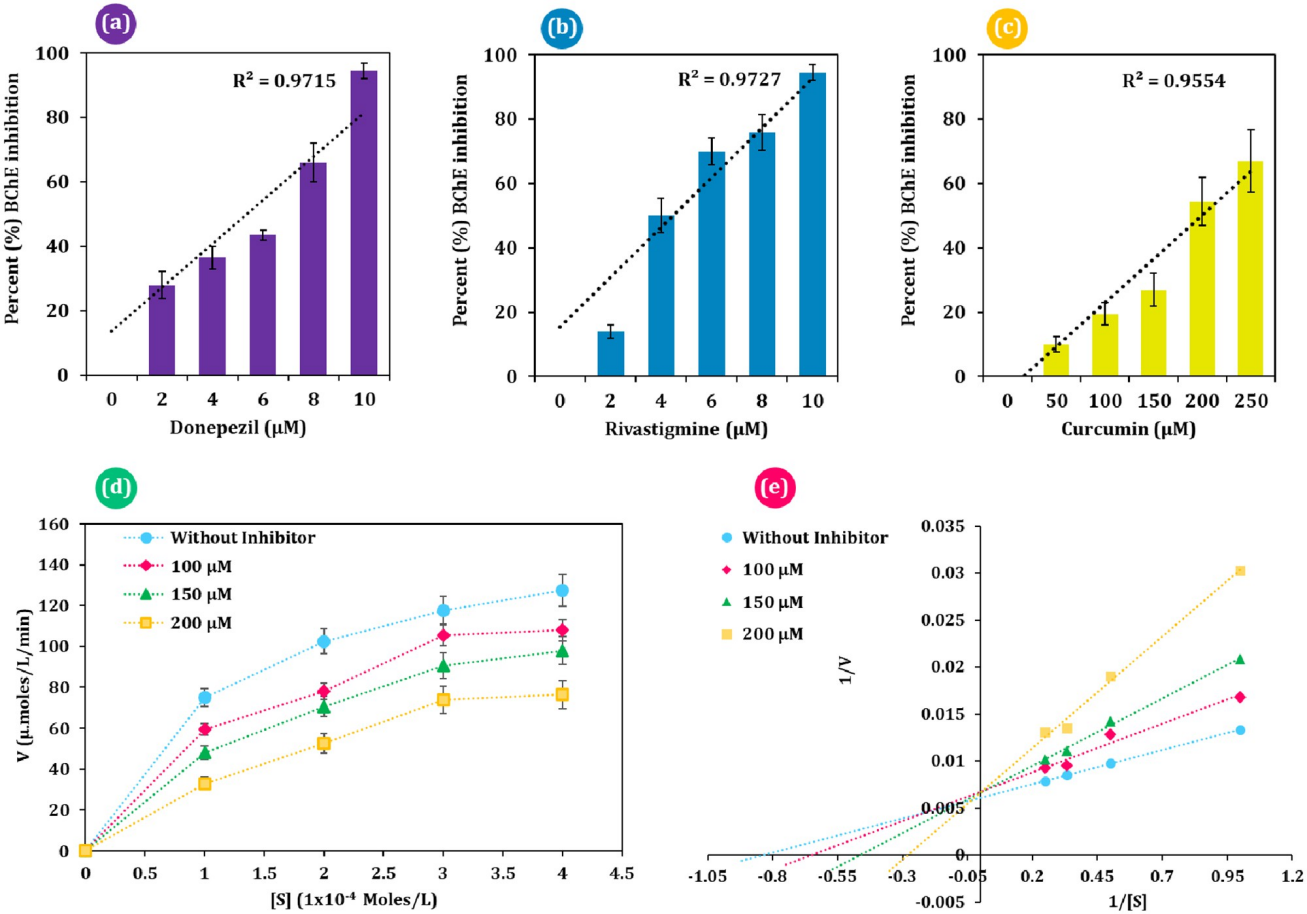
BChE enzyme inhibition assay results, (a) percent BChE inhibition by donepezil, (b) percent BChE inhibition by rivastigmine, (c) percent BChE inhibition by curcumin, (d) Michaelis–Menten (MM) plot with different inhibitor (curcumin) concentration (for a–e; n = 4, error bars, standard error of mean) and (e) double reciprocal Lineweaver-Burke plot representation of the MM plot.

BChE enzyme inhibition was performed from the *Ae*. *aegypti* larval lysate, where donepezil, rivastigmine and curcumin were tested to access inhibition of this enzyme (Fig 13). Donepezil (Fig 13a) and rivastigmine (Fig 13b) showed to inhibit larval BChE up to 90% at the 10 μM concentration. Both the reference drugs demonstrated BChE inhibition in a linear trend and represented direct corelation between dosage of reference drug and percent BChE inhibition. The effective working range of both these drugs were found to between 2 to 10 μM for *Ae*. *aegypti* BChE. Whereas, the effective inhibitory concentrations for curcumin (Fig 13c) was found to be higher with respect to the reference inhibitors, ranging from 50 to 250 μM. The effectivity of curcumin at higher concentration was expected but the noteworthy remark of this experiment is that curcumin can inhibit BChE following same trend as that exhibited by reference inhibitors. IC_50_ values for curcumin, donepezil and rivastigmine was found to be 201.28 μM, 5.68 μM and 4.97 μM respectively.

Consequently, it is important to determine the manner of BChE enzyme inhibition by curcumin; either competitive, uncompetitive, or non-competitive. *In vitro* BChE inhibition enzyme assays with varied doses of curcumin were used to assess the relationship between substrate concentration and rate of enzyme activity. Fig 13d shows the results of this experiment as an enzyme activity against substrate concentration plot, and the same data is represented using a double reciprocal Lineweaver–Burke plot (Fig 13e). Each of these plot’s aid in identifying the change in the K_M_ (Michaelis-Menten constant) of the enzyme reactivity when different inhibitor doses are employed for curcumin. For calculating the K_M_ of an enzyme reaction, the Lineweaver–burke plot is more accurate. The ‘–(1/K_M_)’ can be seen in this plot by the intersection of the negative extrapolation with the negative X-axis. As described in Fig 13e it is observed that as the concentration of curcumin increases the ‘–(1/K_M_)’ becomes greater (Fig 13e). Subsequently, it is observed that the K_M_ value rises as the concentration of curcumin rises, which is an attribute of competitive inhibition. Thus, from the experiment it can be inferred that, curcumin can inhibit dipteran BChE enzyme in a competitive nature, which might serve as its ultimate mode of action for larvicidal activity.

## 4. Discussion

Mosquitoes are known to transmit the deadliest diseases known to mankind such as chikungunya, dengue, filariasis, malaria, yellow fever, West Nile virus, etc. Every year 700 million people in Africa, Central and South America, Mexico, Russia, and most of Asia are affected and approximately two million deaths from diseases that are spread by mosquitoes [37]. To control the outgrowth of mosquitoes several vector control strategies have been employed, of which the most prominent strategy is to make use of chemical insecticides and this strategy is being used since 1800s where development of insecticides such as organochlorine compounds, organophosphates, carbamates, pyrethroids and formamidines, etc, was envisaged. Since then, the overuse of insecticides has imposed the adverse implications on the environment, non-target organisms as well as on human health has been observed.

Acephate, chlorpyrifos, bendiocarb, malathion, ethion, famphur, temephos are broadly classified as carbamates and organophosphates are AChE and BChE [38]; DDT and pyrethroids like resmethrin, permethrin or deltamethrin are axonic excitotoxins which act by inhibiting the closure of voltage gated sodium channels in the axonal membranes [39]; methoprene acts by capping juvenile hormone binding protein [40]; avermectin, fipronil, chlordane heptachlor and phenyl pyrazole serve as GABA receptors antagonists [41]. Apart from this, tyrosine hydroxylase (TH) and phenoloxidase (PO) are identified as relatively newer targets for mosquito control, as their inhibition can stop the production of 3,4-dihydroxyphenylalanine (DOPA), which is necessary for cuticle tanning and immune-associated melanisation.

Of all these targets, ChEs are most widely explored, and their inhibition can rapidly induce mortality in mosquitoes. ChEs, both AChE and BChE which have ability to break down Acetylcholine (ACh) and Butyrylcholine (BCh), respectively, during nerve impulse conduction in neurons. Thus, ChE family serves as the main target for developing new insecticides/pesticides, where accumulation of ACh/BCh causes hyperexcitation and then eventually death of insects [14, 15]. Moreover, both types of ChEs are even found to be present in humans and are essential targets for developing medications for inhibiting ChEs in the treatment of Alzheimer’s disease (AD). As the chemical pesticides being persistent in nature are known to be harmful to humans, in recent time researchers have tried to develop biopesticides, where extract of many plants has shown to induce mortality in insects and mosquitoes. Similarly, as part of alternate medications, researchers have found several phytochemicals that can serve to inhibit ChEs in humans and control AD. For instance, phenylethanoids and terpenes isolated from the methanolic extract of *Verbascum xanthophoeniceum* Griseb and *V*. *mucronatum* have shown ChEs inhibition. Phenylethanoids, diterpenes, flavonoids and naphthoquinones from isolated from the *Calceolaria* species are reported as ChEs inhibitors. Ursolic and oleanolic acid from the leaves of *C*. *talcana* are reported to inhibit AChE. Similarly, phytochemicals from *C*. *talcana* and *C*. *integrifolia* are reported to inhibit AChE [14]. On the other hand, there is a loophole where phytochemicals and plant extracts are reported as biopesticides and their mode of action are not known. Shaalan and colleagues have elaborated the potential phytochemicals that possess mosquitocidal potential in their review [42]. Most of these phytochemicals also work at par with the effectivity of commercially available synthetic larvicides, which leads to the promotion and suggestion of use of plant secondary metabolites as natural larvicide.

Computational rational drug designing and other computational approaches for developing inhibitors, drugs and alternate medicines have gained lot of attention owing to high accuracy and robustness [43–48]. Under current study, the top 13 phytochemicals identified using computational screening that showed probable interaction with BChE are curcumin, desmethoxycurcumin, gingerol, asarinin, capsaicin, sesamin, 6-shogaol, rosmarinic acid, piperine, hesperetin, zingiberene, betulinic acid, and ursolic acid. Previous research has shown curcumin and other curcuminoids to inhibit ChEs of humans and are considered as a potential phytochemicals for treating AD [49–53]. Anti-cancer, anti-alzheimer, anti-inflammatory, anti-viral, anti-bacterial, and many more properties till date is been reported to be exhibited by curcumin and therefore is perceived as phytochemical of miracle [54]. There are reports with *in silico* assessments for curcumin to interact with human AChE [55, 56]. Previously we have reported curcumin to induce mortality in *Cx*. *pipiens* by inhibiting AChE. Further, curcumin and other curcuminoids are well known to impart larvicidal, mosquitocidal and mosquito repellent properties, however the mode of action of these compounds are not completely well understood [57–60]. The next hit, gingerol, is also reported to be an AChE inhibitor [61]. Capsaicin, a phytochemical is reported to be AChE inhibitor [62]. Rosmarinic acid is a polyphenol found in multiple aromatic plants and is reported to inhibit glutathione S-transferase, lactoperoxidase, AChE, BChE and carbonic anhydrase isoenzymes [63]. The next hit, piperine is known to inhibit ChEs, moreover curcumin and piperine are reported to synergically inhibit AChE and BChE in humans [64]. Sesamin is a phytochemical found in *Cortex Acanthopanacis radicis*, is reported to inhibit AChE, and known to improve memory impairment in mouse [65]. Lastly as represented earlier, ursolic and oleanolic acid from the leaves of *C*. *talcana* are reported to inhibit AChE [14]. Majority of the hits obtained from primary screening of docking are known to inhibit AChE in humans if not all in insects. Moreover, all these 13 top hits so obtained had pharmacophore features identical to that of reference drugs donepezil and rivastigmine used in the current study. Pharmacophore mapping is a method for identifying the similar ligands to the reference drug from the database [66]. To consolidate the findings of docking the top hit, curcumin was chosen for further assessment, where MD simulations further provided the robust assessment of interaction of curcumin with BChE, as MD simulations provide best possible near to perfect predictions of ligand protein interactions [47]. To the best of our knowledge this is an holistic report for curcumin to inhibit the dipteran BChE and the claim is further validated by performing *in vitro* enzyme assay using Ellman’s protocol [14, 62, 67]. Lastly, there are several reports suggesting synergic *in silico* and *in vitro* assessment to be extremely useful in identifying inhibitors for various ChEs of humans [68–71], and we have made use of similar approach for identifying inhibitor of dipteran BChE which can have extensive potential to serve as natural larvicidal agent.

## Supporting information

S1 File (DOCX)

S1 Graphical abstract (TIF)
