# Supplementary material for: Extending the lore of curcumin as dipteran Butyrylcholine esterase (BChE) inhibitor: A holistic molecular interplay assessment
Source: PLoS One. 2022 May 26;17(5):e0269036. doi: 10.1371/journal.pone.0269036 (PMC9135230; doi:10.1371/journal.pone.0269036)
Supplement: S1 File — (DOCX) [file pone.0269036.s001.docx]

**Supplementary material file**


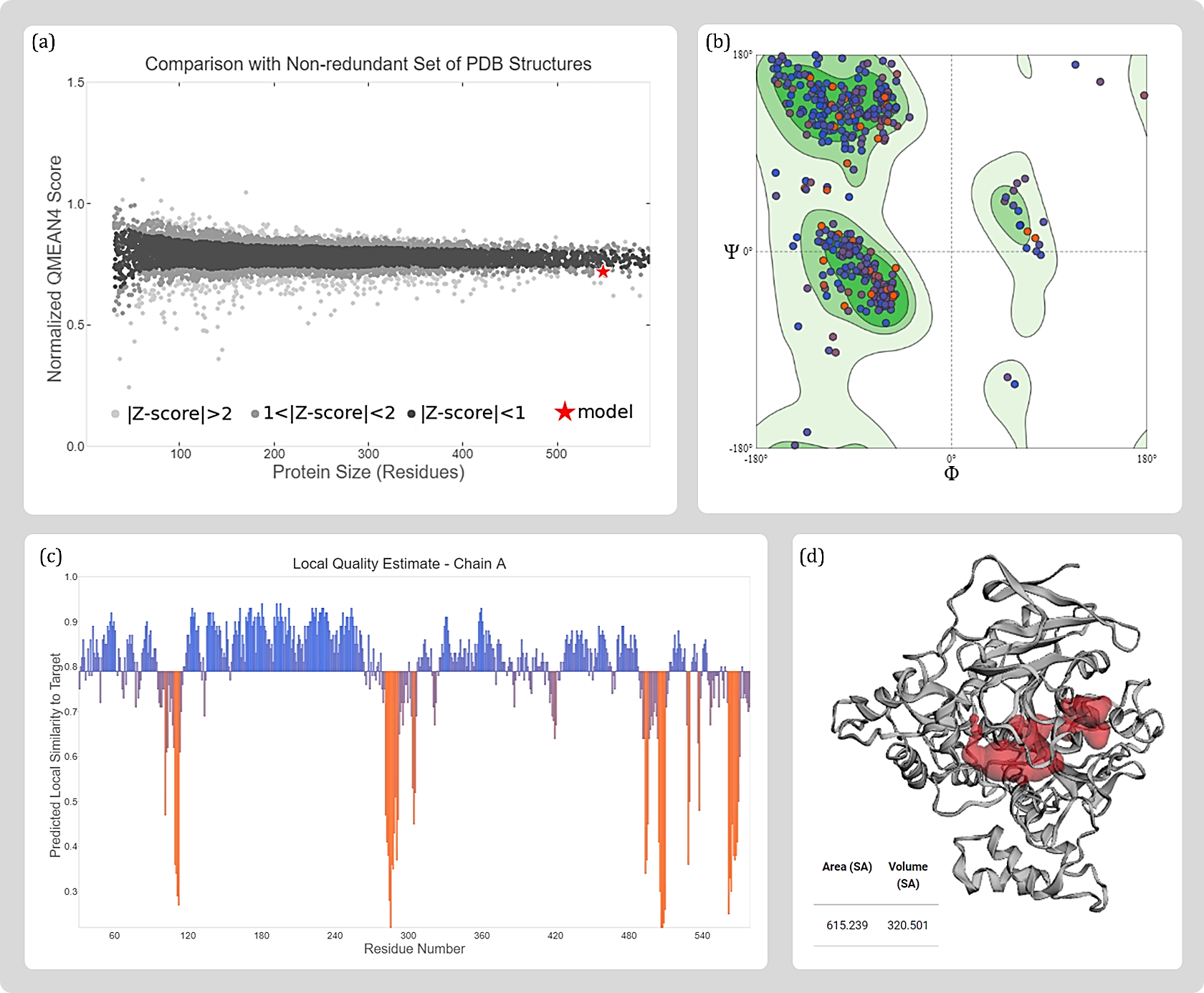


**Figure S1** Quality estimate parameters for modelled BChE protein (a) comparison with non-redundant set of PDB structures (b) Ramachandran plot (c) local model quality estimate and (d) binding pocket identification of modelled protein predicted by CASTp 3.0


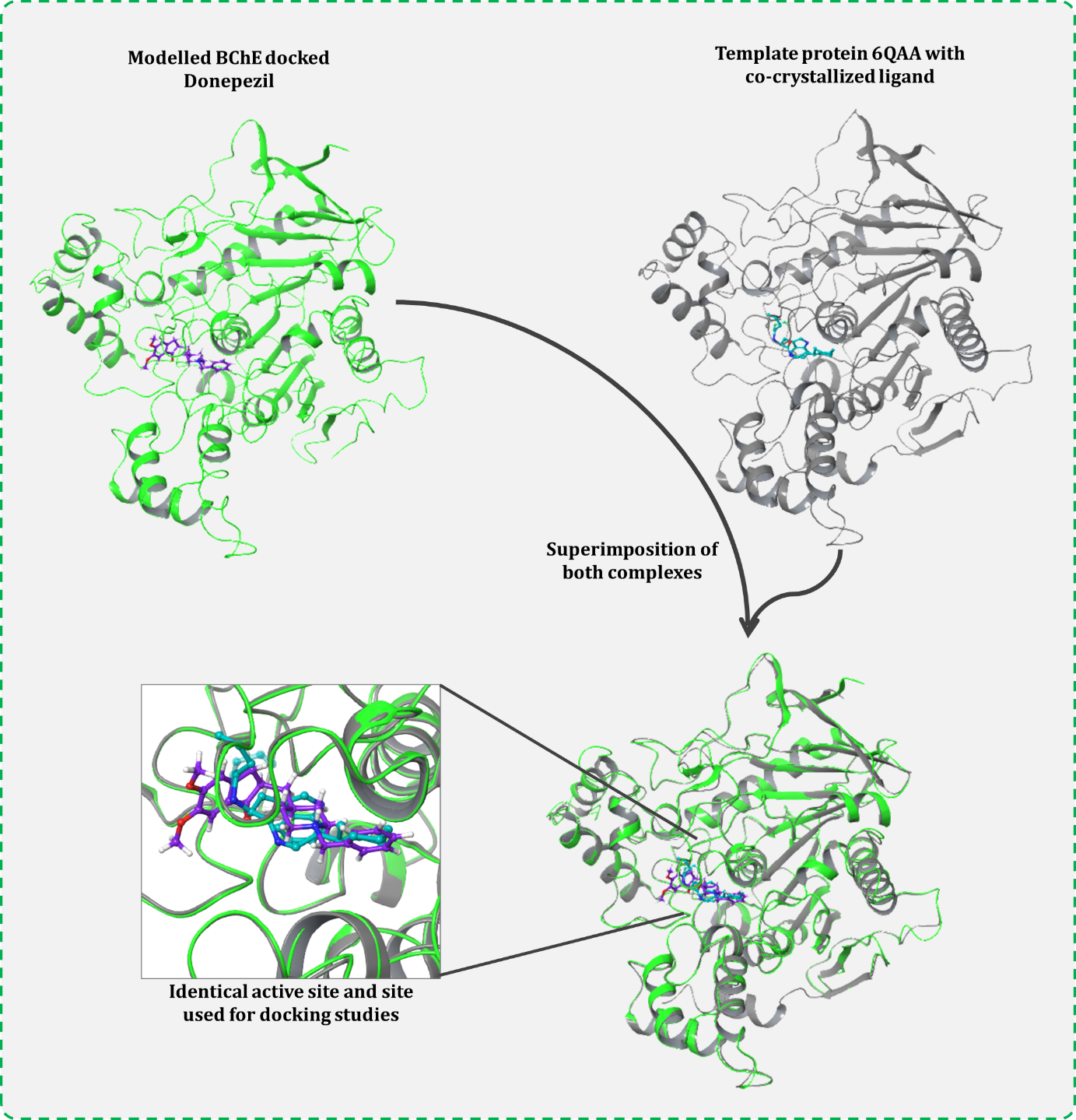


**Figure S2** Validation of correct active site coordinated for docking, proved by identical binding of Donepezil with modelled BChE to that of native ligand of template protein (PDB id 6QAA). Where superimposing both the protein, the active sites also get super imposed, and the respective ligand of proteins also gets superimposed at identical locus. Thus, proving the coordinates of docking used for modelled BChE is apt.
