# Supplementary figures and images for: Extending the lore of curcumin as dipteran Butyrylcholine esterase (BChE) inhibitor: A holistic molecular interplay assessment
Source: PLoS One. 2022 May 26;17(5):e0269036. doi: 10.1371/journal.pone.0269036 (PMC9135230; doi:10.1371/journal.pone.0269036)

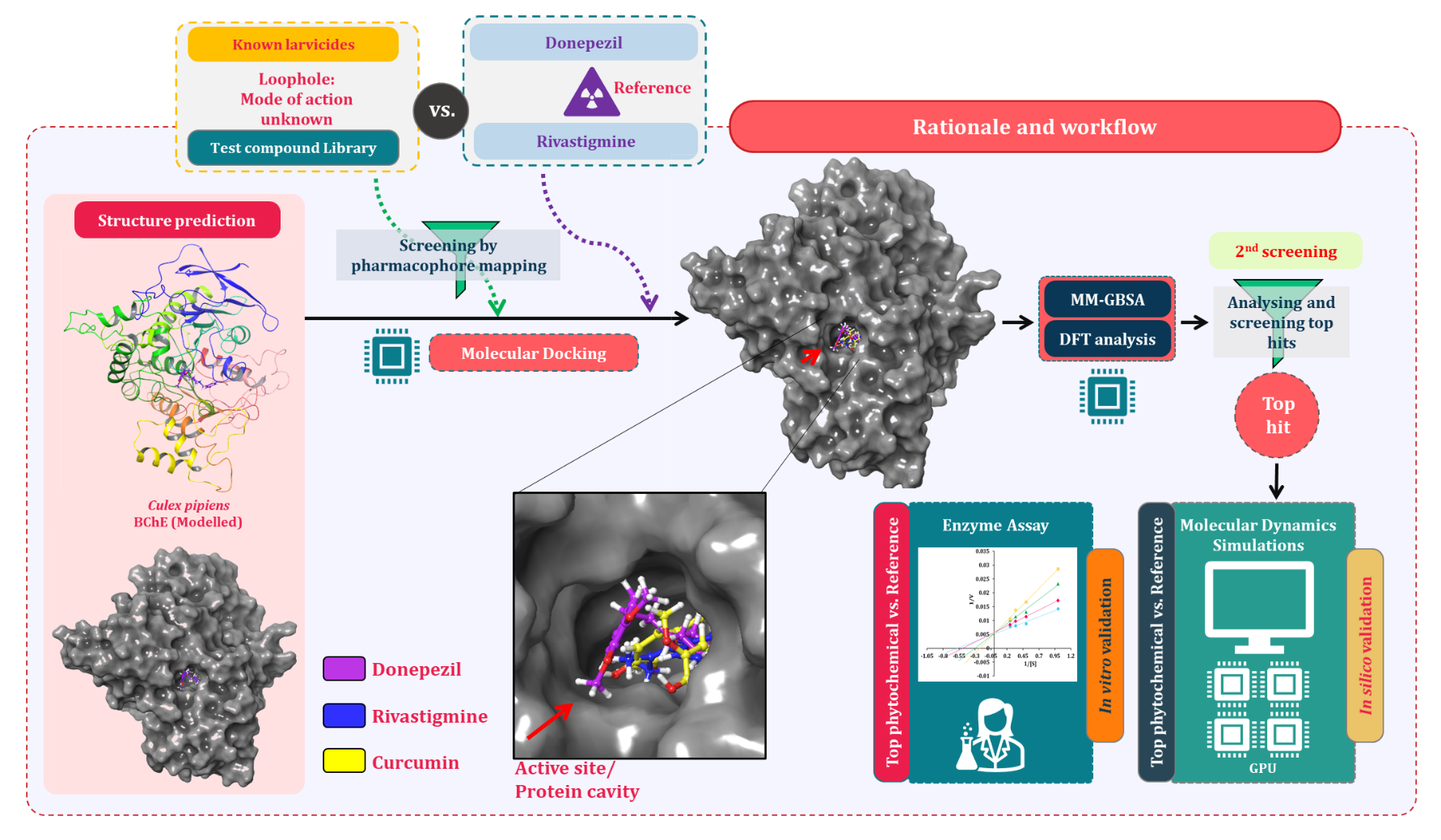

Supplement: S1 Graphical abstract — (TIF) [file pone.0269036.s002.tif]
